# *ATM *variants and cancer risk in breast cancer patients from Southern Finland

**DOI:** 10.1186/1471-2407-6-209

**Published:** 2006-08-16

**Authors:** Johanna Tommiska, Laila Jansen, Outi Kilpivaara, Hege Edvardsen, Vessela Kristensen, Anitta Tamminen, Kristiina Aittomäki, Carl Blomqvist, Anne-Lise Børresen-Dale, Heli Nevanlinna

**Affiliations:** 1 Department of Obstetrics and Gynecology, Helsinki University Central Hospital (HUCH), Helsinki, Finland; 2 Department of Genetics, Institute for Cancer Research, Rikshospitalet-Radiumhospitalet Medical Centre, Oslo, Norway; 3 Medical Faculty, University of Oslo, Oslo, Norway; 4 Department of Clinical Genetics, HUCH, Helsinki, Finland; 5 Department of Oncology, HUCH, Helsinki, Finland; 6 Department of Oncology, Uppsala University Hospital, Sweden

## Abstract

**Background:**

Individuals heterozygous for germline *ATM *mutations have been reported to have an increased risk for breast cancer but the role for *ATM *genetic variants for breast cancer risk has remained unclear. Recently, a common *ATM *variant, *ATMivs38 *-8T>C in cis with the *ATMex39 *5557G>A (D1853N) variant, was suggested to associate with bilateral breast cancer among familial breast cancer patients from Northern Finland. We have here evaluated the 5557G>A and ivs38-8T>C variants in an extensive case-control association analysis. We also aimed to investigate whether there are other *ATM *mutations or variants contributing to breast cancer risk in our population.

**Methods:**

Two common *ATM *variants, 5557G>A and ivs38-8T>C, previously suggested to associate with bilateral breast cancer, were genotyped in an extensive set of 786 familial and 884 unselected breast cancer cases as well as 708 healthy controls. We also screened the entire coding region and exon-intron boundaries of the *ATM *gene in 47 familial breast cancer patients and constructed haplotypes of the patients. The identified variants were also evaluated for increased breast cancer risk among additional breast cancer cases and controls.

**Results:**

Neither of the two common variants, 5557G>A and ivs38-8T>C, nor any haplotype containing them, was significantly associated with breast cancer risk, bilateral breast cancer or multiple primary cancers in any of the patient groups or subgoups. Three rare missense alterations and one intronic change were each found in only one patient of over 250 familial patients studied and not among controls. The fourth missense alteration studied further was found with closely similar frequencies in over 600 familial cases and controls.

**Conclusion:**

Altogether, our results suggest very minor effect, if any, of *ATM *genetic variants on familial breast cancer in Southern Finland. Our results do not support association of the 5557G>A or ivs38-8T>C variant with increased breast cancer risk or with bilateral breast cancer.

## Background

The ATM (Ataxia-Telangiectasia Mutated) kinase has an essential role in maintaining genomic integrity. It is a key activator of the cellular responses to DNA double-strand breaks [1]. Mutations in the *ATM *gene cause ataxia-telangiectasia (A-T) [2], a rare recessive disorder characterized by progressive neurodegeneration, cell cycle checkpoint defects, radiosensitivity and increased risk of cancer, particularly of lymphoid malignancies [3]. As radiation exposure is associated with an increased risk of breast cancer, the function of the ATM protein makes it a good candidate for a role in breast cancer predisposition [4]. The first suggestion that *ATM *might be a breast cancer susceptibility gene came from studies reporting an increased breast cancer risk among obligate heterozygous mutation carriers in A-T families [5,6]. An increased risk for malignancy, in particular, female breast cancer, among individuals heterozygous for germline *ATM *mutations has been reported in many studies [7,8], but in a recent study, the increased breast cancer risk of the heterozygous mutation carriers in A-T families was seen only in the mothers of the A-T patients [9], while another study reported that the breast cancer risk in A-T families was associated specifically with mutations located in the binding domains of the ATM protein [10]. A number of studies have searched for germ line *ATM *mutations in breast cancer cases and/or compared the frequency of common variants among breast cancer cases to population controls, but the evidence regarding the role of *ATM *as a breast cancer susceptibility gene has been contradictory. Overall, the frequency of *ATM *mutations found in breast cancer patients in the general population has been low, and many of the *ATM *variants found are too rare to be evaluated easily in case-control studies. Most of the studies have also been too small to detect modest increases in risk for the common *ATM *variants, (reviewed in [4]). The study of 270 Austrian breast and ovarian cancer families reported a significant prevalence of *ATM *mutations in these families [11], whereas no *ATM *mutations associated with increased breast cancer risk were found in 121 breast or breast-ovarian cancer families from Northern Finland [12]. However, a common variant, *ATMivs38 *-8T>C in cis with the *ATMex39 *5557G>A (D1853N) variant, was suggested to associate with bilateral breast cancer[12]. The 5557G>A variant has previously been reported in the homozygous state to associate with enhanced clinical radiosensitivity in breast cancer patients [13].

In this study, we have evaluated the 5557G>A and ivs38-8T>C variants, suggested to associate with bilateral breast cancer[12], for breast cancer risk and association with bilateral breast cancer or multiple cancers in an extensive set of 786 familial and 884 unselected Finnish breast cancer cases and 708 healthy controls. We further screened the entire coding region and exon-intron boundaries of the *ATM *gene from 47 familial breast cancer patients, in order to determine the *ATM *haplotypes among the Finnish breast cancer patients, and to investigate whether there are other *ATM *mutations or variants contributing to breast cancer risk in Southern Finland.

## Methods

### Breast cancer patients and controls

We evaluated the common polymorphisms *ATMex39 *5557G>A (D1853N) and *ATMivs38 *-8T>C for breast cancer risk by genotyping those in a series of 786 familial breast cancer patients, 884 unselected breast cancer cases and 708 healthy female control subjects from the same geographical region.

The familial breast cancer patients have been collected by a systematic interview for family history at the Helsinki University Central Hospital as previously described [14]. Of the 786 familial patients, the 284 patients (including the 47 breast cancer patients screened for the whole gene and the 237 cases studied also for the rare variants below) have a stronger family history (three or more first or second degree relatives with breast or ovarian cancer in the family, including the proband), as verified through the Finnish Cancer Registry and hospital records, and 502 breast cancer cases (368 of which were also studied for *ATMex10 *998C>T (S333F)) have reported only a single affected first-degree relative. *BRCA1 *and *BRCA2 *mutations have been excluded for all the familial patients with the strong family history of cancer as well as for 291 cases with one affected relative as previously described [15-17]. The series of 884 unselected breast cancer patients studied were collected at the Department of Oncology, Helsinki University Central Hospital during April 1997-March 1998 (described in detail in [18]) and January-June 2000 [19] and cover 79% of all consecutive, newly diagnosed breast cancer cases during the collection periods.

The index patients from forty-seven *BRCA1/2*-negative breast cancer or breast-ovarian cancer families (three or more first or second degree relatives with breast or ovarian cancer in the family, including the proband) were also fully screened for germline variants throughout the coding regions and splice sites of the *ATM *gene. Thirteen of these 47 index patients also had a family history of leukemia and/or lymphoma.

The frequencies of five rare *ATM *germline variants identified (four missense alterations and one intronic change known to lead to incorrect splicing of the *ATM *transcript [20]) were further studied in an additional series of 237 index patients from non-*BRCA1/2 *breast cancer families (three or more first or second degree relatives with breast or ovarian cancer in the family, including the proband) and 237 healthy population controls. One of the five rare variants, *ATMex10 *998C>T (S333F) residing on the same haplotype with the 5557G>A and ivs38-8T>C variants, was then further screened in additional 368 breast cancer cases from families with two affected first-degree relatives, and in 367 healthy controls.

All specimens were collected and analyzed with informed consent and under protocols approved by ethics committees of the Departments of Obstetrics and Gynecology, and Oncology, and the Ministry of Social Affairs and Health in Finland.

### Mutation analyses

The three variants (5557G>A, ivs38-8T>C and 998C>T) were genotyped in extensive patient series and population controls described above, using minisequencing (primer extension [21]), Amplifluor™ fluorescent genotyping (KBiosciences, Cambridge, UK [22]) or restriction fragment length polymorphism analysis (RFLP): the ivs38-8T>C change creates a restriction site for *Rsa*I (New England BioLabs, Beverly, MA, USA); for 998C>T a mutagenesis PCR-primer was designed that creates a restriction site for *Eco*RI (New England BioLabs, Beverly, MA, USA), which is abolished by the 998C>T change. Carriers of the ivs38-8T>C or 998C>T change could be detected in a 2% or 3% agarose gel after *Rsa*I or *Eco*RI digestion of the PCR-products, respectively. All the samples found positive for 998C>T with RFLP were confirmed by direct sequencing. For the ivs38-8T>C variant, all the positive cases were re-analysed by RFLP and a sample of them were also confirmed by direct sequencing. For the 5557G>A variant, 50 samples were genotyped by two independent methods (minisequencing and Amplifluor™ fluorescent genotyping). All the re-analysis and confirmation results were consistent.

Genomic DNA from the 47 familial breast cancer patients was screened for germline alterations in all coding exons and exon-intron boundaries of *ATM *gene by denaturating high-performance liquid chromatography [23] (dHPLC, WAVE, Transgenomic Inc., Omaha, NE, USA). All samples with an abnormal curve were re-amplified and sequenced by direct sequencing using BigDye Terminator Cycle Sequencing Kit and ABI 310 automated sequencer (Applied Biosystems, Foster City, CA, USA). Five rare variants found were further screened by dHPLC and all positive samples were verified by direct sequencing.

### Bioinformatic and statistical analyses

Haplotypes were reconstructed using PHASE (version 2.1.1) [24]. The software implements a Bayesian statistical method for reconstructing haplotypes based on population genotype data [25,26]. SIFT-analysis [27,28] and PolyPhen [29,30] were used to evaluate functional significance of the *ATM *missense variants found. SIFT program calculates tolerance scores for amino acid changes based on sequence alignment and conservation across protein family or across evolutionary history. PolyPhen (=Polymorphism Phenotyping) predicts possible impact of an amino acid substitution on the structure and function of a human protein using straightforward physical and comparative considerations. The differences in variant frequencies were analysed by Fisher's exact test (SPSS version 12.0 for Windows, SPSS Inc., Chicago, IL, USA). All p-values are two-sided, and due to multiple testing, p-value <0.01 was considered significant. Estimates of statistical power and ideal sample size were obtained using the UCLA Department of Statistics power calculator [31,32] and the SISA statistics calculator [33,34].

## Results

The genotype distribution of the common *ATMex39 *5557G>A (D1853N) polymorphism studied was closely similar in breast cancer cases and healthy controls, suggesting no effect on breast cancer risk (Table 1.). No association of this polymorphism with bilateral breast cancer or multiple cancers (breast cancer and at least one other non-breast cancer) was seen among the familial or unselected breast cancer cases (Table 2.). No association with histopathologic features (tumor histology, grade, hormone receptor status, TNM stage) of the breast tumors or survival among the unselected breast cancer patients was seen, either (data not shown). This alteration also appeared well tolerated in SIFT (score 0.17) and PolyPhen (benign) analysis.

No significant effect on breast cancer risk of the *ATMivs38 *-8T>C polymorphism was observed (Table 1.). It was not associated with any histopathologic features of the breast tumors or survival among unselected breast cancer patients (data not shown). The ivs38-8T>C polymorphism was not significantly associated with the risk of bilateral breast cancer among unselected or familial breast cancer patients. Among the unselected breast cancer patients, the ivs38-8T>C polymorphism tended to be associated with an increased risk of multiple primary cancers (breast cancer and at least one other non-breast cancer) (OR 2.56, 95% CI 1.23–5.35, p = 0.02, Fisher's exact test, Table 2.), but the result did not reach statistical significance and was not seen among the familial cases.

*ATM *sequence variants found in the full gene screen from 47 Finnish familial breast cancer patients as well as haplotypes constructed with PHASE software are presented in Table 3. Altogether 17 different sequence variants forming 17 different haplotypes were found. Seven of the variants were intronic, four were silent nucleotide substitutions and six were missense changes.

The 5557G>A variant was present in altogether six haplotypes (haplotypes 7, 8, 9, 10, 12 and 13 in Table 3.), and three of them (haplotypes 8, 9 and 13 in Table 3.) contained also the ivs38-8T>C variant. The haplotype 8 contains only the combined variant 5557G>A, ivs38-8T>C, whereas the haplotype 13 contains also the missense variant 998C>T (S333F) in exon 10. To further evaluate this haplotype the missense variant 998C>T was studied in additional 368 breast cancer cases with a moderate family history and in 367 healthy controls, as well as in 237 breast cancer cases with a stronger family history and in 237 healthy controls (see below). The third haplotype (haplotype 9) containing the combined variant 5557G>A, ivs38-8T>C included also the silent change *ATMex32 *4578C>T (P1526P), which was found in only 1/47 of our fully screened cases.

Four rare missense alterations and one of the intronic changes, *ATMivs10 *-6T>G, a known A-T mutation leading to skipping of the exon eleven in the transcript [20], were screened in additional series of 237 familial breast cancer cases and 237 healthy controls. The frequencies of these five variants in breast cancer cases and healthy controls are presented in Table 4. None of the variants segregated with cancer in the families studied. Only the index case with 998C>T (S333F) carried the variant in one family studied, with three other affected sisters being non-carriers; for two other families no additional samples were available. Similarly, only the index cases carried the 1814A>G (H605R) and ivs10-6T>G variants. For 1814A>G, three other affected relatives (sister, cousin and her daughter) were not carriers. For the ivs10-6T>G variant, the two other affected in the family were not carriers whereas two of the three healthy sisters (aged 63 and 73 years) of the index case carried the variant. Unfortunately, no samples of affected relatives were available for the 4424A>G (Y1457C) and 6539G>T (G2180V) variant carrier cases. The fifth rare missense alteration, *ATMex15 *2119T>C (S707P), was not studied further as it has been extensively studied previously [20,35-37], and the results do not support its association with breast cancer.

## Discussion

The common polymorphism, *ATMex39 *5557G>A (D1853N), has been reported in the homozygous state to associate with enhanced clinical radiosensitivity in breast cancer patients [13], suggesting that it might be considered as a risk factor predisposing to adverse reactions after radiotherapy and supporting a possible functional effect for this variant. Heikkinen et al. [12] recently reported that the *ATMivs38 *-8T>C polymorphism occurring in cis position with 5557G>A was associated with bilateral breast cancer, among altogether 176 familial breast cancer patients studied. The combined variant was found to associate also with reduction of ATM protein level in lymphoblast cells. No aberrant transcripts were detected [12], although it was hypothesized that the 5557G>A variant previously suggested to affect an exonic splicing enhancer element [11] together with the ivs38-8T>C change could have some effect on the correct splicing of the exon 39. Most recently, Langholz et al. [38] reported that the association of ivs38-8T>C with bilateral breast cancer could not be replicated in the WECARE Study population of 708 asynchronous bilateral and 1397 unilateral breast cancer patients. While this does not support the previously suggested association of the variant with bilateral breast cancer, Langholz et al. [38] discussed the possibility that the variants studied may not be the causative alleles but may be contained in the same risk haplotype with other, possible risk alleles unique to the Finnish population. The WECARE cases were also unselected for family history while those in the study by Heikkinen et al. [12] belonged to breast cancer families. In our study, including about 800 unselected as well as almost 800 familial Finnish breast cancer cases, neither 5557G>A nor ivs38-8T>C, or any haplotype containing these variants in the Finnish population, was associated with breast cancer risk or bilateral breast cancer in any of the patient groups or subgoups studied. Among our familial breast cancer patients screened for the whole *ATM *gene, both of the variants 5557G>A and ivs38-8T>C were present in altogether three haplotypes (haplotypes 8, 9 and 13 in Table 3.). Our results suggest that the haplotype 8, containing only the combined variant 5557G>A, ivs38-8T>C, is not associated with bilateral breast cancer. The haplotype 13 containing also the missense variant 998C>T (S333F) in exon 10 is far too rare to underlie the suggested association with bilateral breast cancer [12]: only 6/652 (0.92%) familial breast cancer patients carried this haplotype. The third haplotype (haplotype 9) containing the combined variant 5557G>A, ivs38-8T>C included also the silent change *ATMex32 *4578C>T (P1526P), which was found in only 1/47 of our fully screened cases. This variant was not suggested to be in linkage disequilibrium with other variants in Northern Finland [12], nor in the HapMap database [39], and it was not suggested to associate with bilateral breast cancer [12]. Although the number of cases studied for determining the haplotypes was only 47 (94 chromosomes), the 95% confidence intervals (0.000 – 0.039) for undetected haplotypes (frequency 0.000) suggest a maximum combined frequency of 3.9% for undetected haplotypes with a 95% probability. This suggests also that any undetected haplotypes would have been rare and unlikely to account for the proposed association of ivs38-8T>C with bilateral breast cancer.

The carrier frequency of the ivs38-8T>C variant (or the combined variant, as all the carriers of ivs38-8T>C also carried 5557G>A) was marginally higher in familial breast cancer patients, especially in those patients with only a moderate family history of breast cancer (8.1%), than in healthy controls (5.6%), but the difference does not reach statistical significance. Given that the case-control tests for the ivs38-8T>C variant yielded positive odds ratios, post hoc calculations were performed to evaluate the statistical power of our analysis. If the observed OR of 1.32 for familial breast cancer risk was assumed to reflect the actual population risk for familial breast cancer, a sample size of 2974 cases and controls would be required to reach 80% power to correctly reject the null hypothesis of no association. The current sample size has only a 29% power to detect a similar effect size. However, our study had 80% power to detect an association with an odds ratio of 1.70 or more among familial cases (with odds ratio 2.86 for bilateral breast cancer and 2.77 for multiple cancer) and 1.68 among unselected cases (with odds ratio 3.6 for bilateral breast cancer and 2.9 for multiple cancer), at a significance threshold of 0.05. Our results do not support an association of the variants with increased breast cancer risk in any of the patient groups studied, however, small increases in risk cannot be excluded. However, our results are consistent with those by Langholz et al. [38] who also found no association of the 5557G>A/ivs38-8T>C variant with bilateral breast cancer (OR = 1, 95% CI 0.6–1.5). The variants did not associate (together or alone) with histopathologic features of the tumors, survival of the patients or the age of first breast cancer diagnosis among the unselected breast cancer patients, either (data not shown).

As A-T patients have an increased risk for lymphoid malignancies, we also evaluated whether the 5557G>A and ivs38-8T>C variants (together or alone) associate with family history of these malignancies. No such effect was seen (data not shown). The variants were not significantly associated with multiple primary tumors, either.

Screening of the whole coding region of the gene revealed altogether 17 different sequence variants in the *ATM *gene in the 47 familial breast cancer patients. Seventeen different haplotypes were also seen, with haplotype frequency 0.65 for the major haplotype 1 with no *ATM *variation present in the exonic and adjacent intronic regions studied. The two most common haplotypes (with frequencies of 0.65 and 0.15) represented 80% of the haplotypes of the patients studied here, and the three most common haplotypes (the third with a frequency of 0.03) accounted for 83% of all the haplotypes here. The haplotype distribution in the *ATM *locus in the Finnish population seems similar to other populations studied, where two to three major *ATM *haplotypes representing >80% of the haplotypes have been identified and suggested to explain the majority of *ATM *variation [13,40,41]. However, direct comparison of the actual haplotypes is difficult as different markers have been used in different studies for evaluating the haplotypes, including also non-coding single nucleotide polymorphisms (SNPs) in and around the *ATM *locus. *ATM *haplotypes have been also reported to associate with increased breast cancer risk in Korean population [42]; in that study, one coding and four intronic SNPs were used for haplotype construction. The International HapMap Consortium [39,43] has genotyped 298 SNPs in the *ATM *containing region on chromosome 11, including five of the SNPs identified here; *ATMex9 *735C>T (V245V), *ATMex15 *2119T>C (S707P), *ATMex32 *4578C>T (P1526P), *ATMivs38 *-8T>C and *ATMex39 *5557G>A (D1853N). When applying the criteria used by the HapMap project in determining tag-SNPs (r^2^>0.80), only one of the SNPs, 5557G>A, is in complete linkage disequilibrium (LD) with other SNPs genotyped by the HapMap project; rs3092991 and rs4988023 located in the middle of the intron 20 and 35, respectively.

Of the 17 variants identified in this study, seven were intronic, four were silent and six were missense changes. All the intronic and silent changes have been reported previously. One of the silent changes, *ATMex9 *735C>T (V245V), has previously been found to co-occur with skipping of exon 9 in some A-T patients [44]. However, other causative A-T mutations have more recently been identified in these families (Børresen-Dale, unpublished). In addition, the reported skipping of exon 9 was detected only on cDNA and is seen occasionally probably because the degradation of non-proofread transcripts has not occurred, or it may be an artifact caused by mRNA instability (Børresen-Dale, unpublished). This silent change was also reported to be more frequent in controls than in breast cancer cases from Northern Finland [12]. Based on these, this variant was not screened further in our study. One of the intronic changes, ivs10-6T>G, a known A-T mutation leading to incorrect splicing of the exon 11 and premature truncation of the protein [20], has been suggested to associate with breast cancer in different populations [11,45,46], but larger case-control studies have not found a significant difference in frequencies between breast cancer cases and healthy controls [47-49]. In our study, ivs10-6T>G was found in one of 265 familial breast cancer cases and not in 228 controls and did not segregate with cancer in the family.

One of the five rare missense variants identified, 2119T>C (S707P), has been very extensively evaluated for breast cancer risk previously [20,35-37], and the results do not support its association with breast cancer. All the four rare missense variants studied further, 1814A>G (H605R), 4424A>G (Y1457C), 6539G>T (G2180V) and 998C>T (S333F), may affect ATM function as suggested by bioinformatic analysis (Table 4.). Three of these variants were very rare, each identified in only one out of >250 cases while not in population controls. The 1814A>G variant has previously been found also in a Danish breast cancer patient (Børresen-Dale, unpublished), whereas the 4424A>G variant has been found in a healthy population control but not among breast cancer patients [36]. To our knowledge, the 6539G>T variant has not been reported previously. Even if having functional effect on the ATM protein and possibly being pathogenic, the rarity of these variants limits any potential contribution to breast cancer susceptibility. The 998C>T was found in 0.92% of the cases and in 0.83% of the controls, not supporting an association with breast cancer. We have previously investigated whether the seven A-T mutations found in Finnish A-T families [44,50] are seen also among familial breast cancer patients from Southern Finland. Only one of these mutations, *ATMex53 *7570G>C (A2524P), was found in one case among the 373 studied familial breast cancer patients [53]. Altogether, these results suggest that possible breast cancer associated *ATM *mutations are very rare in breast cancer families from Southern Finland. However, whether rare *ATM *mutations, estimated to be present in about 0.4%–1% of the population [8,51], confer an increased breast cancer risk in the population, is beyond the scope of this study and has not been evaluated at a large scale in other studies yet either. Due to the rarity of such variants, very large population-based case-control studies will be required [52].

## Conclusion

Our results do not support an association of 5557G>A or ivs38-8T>C variant, or any haplotype containing these in the Finnish population, with bilateral breast cancer or with increased breast cancer risk. Altogether, our results suggest very minor effect, if any, of *ATM *genetic variants on familial breast cancer in Southern Finland.

## Competing interests

The author(s) declare that they have no competing interests.

## Authors' contributions

JT carried out most of the molecular genetic studies, performed the statistical analyses and drafted the manuscript. LJ participated in the dHPLC analyses. OK and AT assisted in the molecular genetic studies. HE and VK constructed the haplotypes. KA and CB collected the patient samples and clinical data in the study. ALBD and HN participated in the study design and helped to draft the manuscript.

## Pre-publication history

The pre-publication history for this paper can be accessed here:



## Figures and Tables

**Table 1:** *ATMex39 *D1853N and *ATMivs38*(-8)T>C genotype frequencies among population controls and unselected and familial breast cancer patients, by family history

***ATMex39 *D1853N**	total	DD		DN		NN		OR (95% CI)*	p
population controls	702	404	57.5%	260	37.0%	38	5.4%		
unselected breast cancer patients	803	485	60.4%	285	35.5%	33	4.1%	0.89 (0.72–1.09)	0.27
familial breast cancer patients	778	469	60.3%	276	35.5%	33	4.2%	0.89 (0.73–1.10)	0.29
index with one affected first degree relative	502	309	61.6%	170	33.9%	23	4.6%	0.85 (0.67–1.07)	0.17
three or more affected in the family	276	160	58.0%	106	38.4%	10	3.6%	0.98 (0.74–1.30)	0.94
* compared to population controls, DN+NN vs. DD									

***ATMivs38*(-8)T>C**	total	TT		TC		OR (95% CI)	p		

population controls	708	668	94.4%	40	5.6%				
unselected breast cancer patients	818	769	94.0%	49	6.0%	1.06 (0.69–1.64)	0.83		
familial breast cancer patients	774	717	92.6%	57	7.4%	1.32 (0.87–2.02)	0.21		
index with one affected first degree relative	495	455	91.9%	40	8.1%	1.47 (0.93–2.31)	0.10		
three or more affected in the family	279	262	93.9%	17	6.1%	1.08 (0.60–1.95)	0.76		

**Table 2:** Multiple cancer and bilateral breast cancer among unselected and familial breast cancer patients, by *ATMex39 *D1853N and *ATMivs38*(-8)T>C genotype

***ATMex39 *D1853N**	total		DD		DN		NN		OR (95% CI)*	p
**Unselected breast cancer patients**	803		485		285		33			
Bilateral	52	6.5%	37	7.6%	14	4.9%	1	3.0%	0.60 (0.32–1.11)	0.11
Unilateral	751	93.5%	448	92.4%	271	95.1%	32	97.0%		
										
Multiple cancer	83	10.3%	51	10.5%	31	10.9%	1	3.0%	0.95 (0.60–1.52)	0.91
Breast cancer only	720	89.7%	434	89.5%	254	89.1%	32	97.0%		
										
**Familial breast cancer patients**	778		469		276		33			
Bilateral	86	11.1%	53	11.3%	30	10.9%	3	9.1%	0.94 (0.59–1.49)	0.82
Unilateral	692	88.9%	416	88.7%	246	89.1%	30	90.9%		
										
Multiple cancer	89	11.4%	57	12.2%	29	10.5%	3	9.1%	0.84 (0.53–1.32)	0.49
Breast cancer only	689	88.6%	412	87.8%	247	89.5%	30	90.9%		
									*DN+NN vs. DD	

***ATMivs38*(-8)T>C**	total		TT		TC		OR (95% CI)	p		

**Unselected breast cancer patients**	818		769		49					
Bilateral	49	6.0%	45	5.9%	4	8.2%	1.43 (0.49–4.15)	0.53		
Unilateral	769	94.0%	724	94.1%	45	91.8%				
										
Multiple cancer	80	9.8%	70	9.1%	10	20.4%	2.56 (1.23–5.35)	0.02		
Breast cancer only	738	90.2%	699	90.1%	39	79.6%				
										
**Familial breast cancer patients**	774		717		57					
Bilateral	84	10.9%	78	10.9%	6	10.5%	0.96 (0.40–2.32)	1.0		
Unilateral	690	89.1%	639	89.1%	51	89.5%				
										
Multiple cancer	92	11.9%	85	11.9%	7	12.3%	1.04 (0.46–2.37)	0.83		
Breast cancer only	682	88.1%	632	88.1%	50	87.7%				

**Table 3:** *ATM *sequence variants and haplotypes found in 47 Finnish familial breast cancer patients

	**variant**	**ex5**	**ex9**	ex10**	ex11**	ex14**	**ex15**	**ex20**	ex31**	**ex32**	**ex39a**	ex39b***	ex39c***	**ex40**	**ex41**	**ex46**	ex47**	**ex62**
	
	**freq**.	1/47	5/47	1/47	1/47	1/47	1/47	1/47	1/47	1/47	1/47	4/47	18/47*	1/47	1/47	2/47	1/47	2/47
**freq**.	**haplotype**																	
0.65	**1**	T	C	C	T	A	T	-	A	C	G	T	G	G	T	T	G	A
0.01	**2**	T	C	C	T	A	T	-	A	C	G	T	G	G	T	T	G	**C**
0.01	**3**	T	C	C	T	A	T	-	A	C	G	T	G	G	T	T	**T**	A
0.02	**4**	T	C	C	T	A	T	-	A	C	G	T	G	G	T	**C**	G	A
0.01	**5**	T	C	C	T	A	T	-	A	C	G	T	G	**A**	T	T	G	**C**
0.01	**6**	T	C	C	T	A	T	-	A	C	**C**	T	G	G	T	T	G	A
0.15	**7**	T	C	C	T	A	T	-	A	C	G	T	**A**	G	T	T	G	A
0.02	**8**	T	C	C	T	A	T	-	A	C	G	**C**	**A**	G	T	T	G	A
0.01	**9**	T	C	C	T	A	T	-	A	**T**	G	**C**	**A**	G	T	T	G	A
0.01	**10**	T	C	C	T	A	T	-	**G**	C	G	T	**A**	G	T	T	G	A
0.01	**11**	T	C	C	T	**G**	T	-	A	C	G	T	G	G	T	T	G	A
0.01	**12**	T	C	C	**G**	A	T	-	A	C	G	T	**A**	G	T	T	G	A
0.01	**13**	T	C	**T**	T	A	T	-	A	C	G	**C**	**A**	G	T	T	G	A
0.01	**14**	T	**T**	C	T	A	T	**A**	A	C	G	T	G	G	**C**	T	G	A
0.03	**15**	T	**T**	C	T	A	T	-	A	C	G	T	G	G	T	T	G	A
0.01	**16**	T	**T**	C	T	A	**C**	-	A	C	G	T	G	G	T	T	G	A
0.01	**17**	**C**	C	C	T	A	T	-	A	C	G	T	G	G	T	T	G	A
															
**variant**	**nt change**	**aa change**	**rs-number**															
															
**ex5**	162T>C	Y54Y	**3218690**															
**ex9**	735C>T	V245V	**3218674**^#^															
**ex10**	998C>T	S333F	**28904919**															
**ex11**	ivs10(-6)T>G	-	-															
**ex14**	1814A>G	H605R	-															
**ex15**	2119T>C	S707P	**4986761**^#^															
**ex20**	ivs20(+28)insA	-	-															
**ex31**	4424A>G	Y1475C	-															
**ex32**	4578C>T	P1526P	**1800889**^#^															
**ex39a**	ivs38(-15)G>C	-	**3092828**															
**ex39b**	ivs38(-8)T>C	-	**3092829**^#^															
**ex39c**	5557G>A	D1853N	**1801516**^#^															
**ex40**	ivs40(+27)G>A	-	**3218686**															
**ex41**	5793T>C	A1931A	**3092910**															
**ex46**	ivs45(-54)T>C	-	-															
**ex47**	6539G>T	G2180V	-															
**ex62**	ivs 62(+8)A>C	-	-															

# these SNPs have been studied in the HapMap project

* two homozygotes

** studied further in series of breast cancer cases and controls, see also Table 4.

*** studied further in series of breast cancer cases and controls, see also Table 1.

**Table 4:** Frequencies of five *ATM *sequence variants in Finnish breast cancer cases and controls

**variant**	**cases**	**controls**	**SIFT**	**PolyPhen**
**ex10**	6/652	5/604		
998C>T (S333F)	0.92%	0.83%	0.00	benign
				
**ex11**	1/265	0/228		
ivs10-6T>G	0.38%	0.00%		
				
**ex14**	1/253	0/232		
1814A>G (H605R)	0.40%	0.00%	0.00	possibly damaging
				
**ex31**	1/273	0/234		
4424A>G (Y1475C)	0.37%	0.00%	0.08	probably damaging
				
**ex47**	1/264	0/234		
6539G>T (G2180V)	0.38%	0.00%	0.03	probably damaging
